# *EIF1AX* Mutations in thyroid nodules and advanced thyroid carcinoma: a systematic review and meta-analysis

**DOI:** 10.1007/s00428-026-04708-7

**Published:** 2026-09-30

**Authors:** Vincenzo Fiorentino, Patrizia Straccia, Esther Diana Rossi

**Affiliations:** 1 Anatomic Pathology Unit, Department of Human Pathology in Adult and Developmental Age “Gaetano Barresi”, University of Messina, Messina, Italy; 2 Division of Anatomic Pathology and Histology, Fondazione Policlinico Universitario “Agostino Gemelli” IRCCS, Rome, Italy; 3 Division of Anatomic Pathology and Histology, Fondazione Policlinico Universitario “Agostino Gemelli” IRCCS, Università Cattolica del Sacro Cuore, Largo Francesco Vito 1, Rome, 00168 Italy

**Keywords:** *EIF1AX*, Thyroid nodules, Bethesda, Molecular testing, Risk of malignancy, Poorly differentiated thyroid carcinoma, Differentiated high-grade thyroid carcinoma, Anaplastic thyroid carcinoma, Meta-analysis

## Abstract

*EIF1AX* alterations occur across benign, borderline, differentiated, and high-grade thyroid neoplasia, requiring contextual interpretation. PubMed/MEDLINE, Scopus, and Embase were searched through 6 May 2026; complementary genomic, citation, and supplementary-data searches continued through 19 July 2026. Analyses addressed prevalence in strict Bethesda III/IV nodules, tissue-verified outcomes in *EIF1AX*-positive Bethesda III/IV nodules, and prevalence in WHO- or Turin-defined poorly differentiated thyroid carcinoma (PDTC) and histology-defined anaplastic thyroid carcinoma (ATC) cohorts not molecularly preselected. Proportions were pooled with binomial-normal generalized linear mixed models; study intervals were exact Clopper-Pearson intervals and heterogeneity was τ² on the logit scale. Among 43 reports, 13 contributed to a primary estimate and 17 to a primary or sensitivity analysis. *EIF1AX* prevalence in three strict Bethesda III/IV cohorts was 4.75% (92/1935; 95% CI, 3.89%–5.80%; τ²=0.000). Malignant histology occurred in 50/117 tissue-verified nodules (40.77%; 95% CI, 24.59%–59.24%; τ²=0.413). Including noninvasive follicular thyroid neoplasm with papillary-like nuclear features (NIFTP)/borderline outcomes and compatible Desai and Connelly data yielded 64/124 (48.16%; 95% CI, 30.69%–66.10%; τ²=0.497). Prevalence was 6.15% in PDTC (11/136; 95% CI, 2.04%–17.12%; τ²=0.455) and 15.06% in ATC (25/162; 95% CI, 9.19%–23.70%; τ²=0.154). *EIF1AX* is uncommon in indeterminate cytology and is not a stand-alone rule-in marker. Interpretation requires variant class, co-alterations, assay, verification, and histologic context.

## Key points

*EIF1AX* prevalence was 4.75% (95% CI, 3.89%–5.80%) in strictly extractable Bethesda III/IV cohorts; the broader source-defined sensitivity was 4.61%.

Among selected *EIF1AX*-positive Bethesda III/IV nodules with tissue verification, the pooled malignant proportion was 40.77% (95% CI, 24.59%–59.24%); this is not a universal preoperative predictive value.

Counting NIFTP/borderline outcomes and the outcome-compatible Desai and Connelly cases produced 48.16% (95% CI, 30.69%–66.10%).

WHO- or Turin-defined PDTC prevalence was imprecise and study dependent (6.15%; 95% CI, 2.04%–17.12%); prevalence in histology-defined ATC cohorts not molecularly preselected was 15.06% (95% CI, 9.19%–23.70%).

Variant-class and co-alteration estimates are descriptive and exploratory because only three or four independent study clusters contributed.

## Introduction

 Molecular testing is increasingly used to refine management of thyroid nodules with indeterminate cytology, particularly Bethesda categories III and IV, in which morphology alone cannot reliably distinguish benign follicular proliferations from low-grade neoplasia. Contemporary cytology-based studies show that assay scope, liquid-based preparation, pre-analytic quality, and institutional workflow influence the diagnostic and prognostic information delivered by molecular testing [1–4].

Because no molecular result is interpreted in isolation, clinical use also depends on ultrasound, cytologic category, sample quality, patient factors, and local thresholds for surgery. Computational decision-support methods have also been explored for indeterminate thyroid nodules, but require external validation and transparent integration with morphology and molecular findings [5].

*EIF1AX* encodes eukaryotic translation initiation factor 1A, a component of the translation pre-initiation complex involved in start-codon selection. Recurrent *EIF1AX* alterations were initially recognized in papillary thyroid carcinoma and were subsequently described in benign and borderline follicular-patterned lesions, indeterminate nodules, follicular and oncocytic carcinomas, and advanced thyroid carcinomas [6–28]. This wide phenotypic distribution prevents a simple rule-in interpretation.

Two molecular dimensions are particularly relevant. First, alterations involving the recurrent A113 splice region have frequently been associated with neoplasia, although estimates are inconsistent. Second, additional alterations—especially *RAS*-family, *TERT* promoter, *TP53*, and other progression-associated events—may identify a biologically different subset from isolated *EIF1AX* [7, 9–13, 21, 22, 29–32]. These observations support a context-dependent working model rather than a deterministic gene-level classification.

The denominator is equally important. Prevalence among all molecularly tested indeterminate nodules, tissue-verified outcome among selected *EIF1AX*-positive nodules, and prevalence in PDTC or ATC answer distinct clinical and biological questions. The review therefore addressed three separate clinical questions, with endpoint-specific eligibility rules applied to preserve consistent cytologic populations and contemporary histologic categories.

## Methods

### Evidence sources and search strategy

A structured search of PubMed/MEDLINE, Scopus, and Embase was performed from database inception to 6 May 2026. The search strategy combined *EIF1AX*, eIF1A, and the full gene name with terms for thyroid nodules, thyroid carcinoma, papillary thyroid carcinoma, follicular thyroid carcinoma, poorly differentiated thyroid carcinoma, anaplastic thyroid carcinoma, Bethesda cytology, fine-needle aspiration, and FNA. Reference lists of eligible studies and relevant reviews were screened manually.

The search identified 155 database records (34 from PubMed/MEDLINE, 63 from Scopus, and 58 from Embase), plus seven records identified through initial reference-list screening. To capture genomic reports in which *EIF1AX* was confined to figures or supplementary data, complementary searches through 19 July 2026 combined a PubMed genomic strategy, backward and forward citation tracking, related-record screening, and systematic review of full texts, oncoprints, and supplementary datasets. Forty-three unique full-text reports were assessed in these complementary searches; 31 met the clinical-report eligibility criteria and 12 were excluded or retained only as mechanistic, technical, case-report, secondary-analysis, or overlap context.

**Fig. 1:**
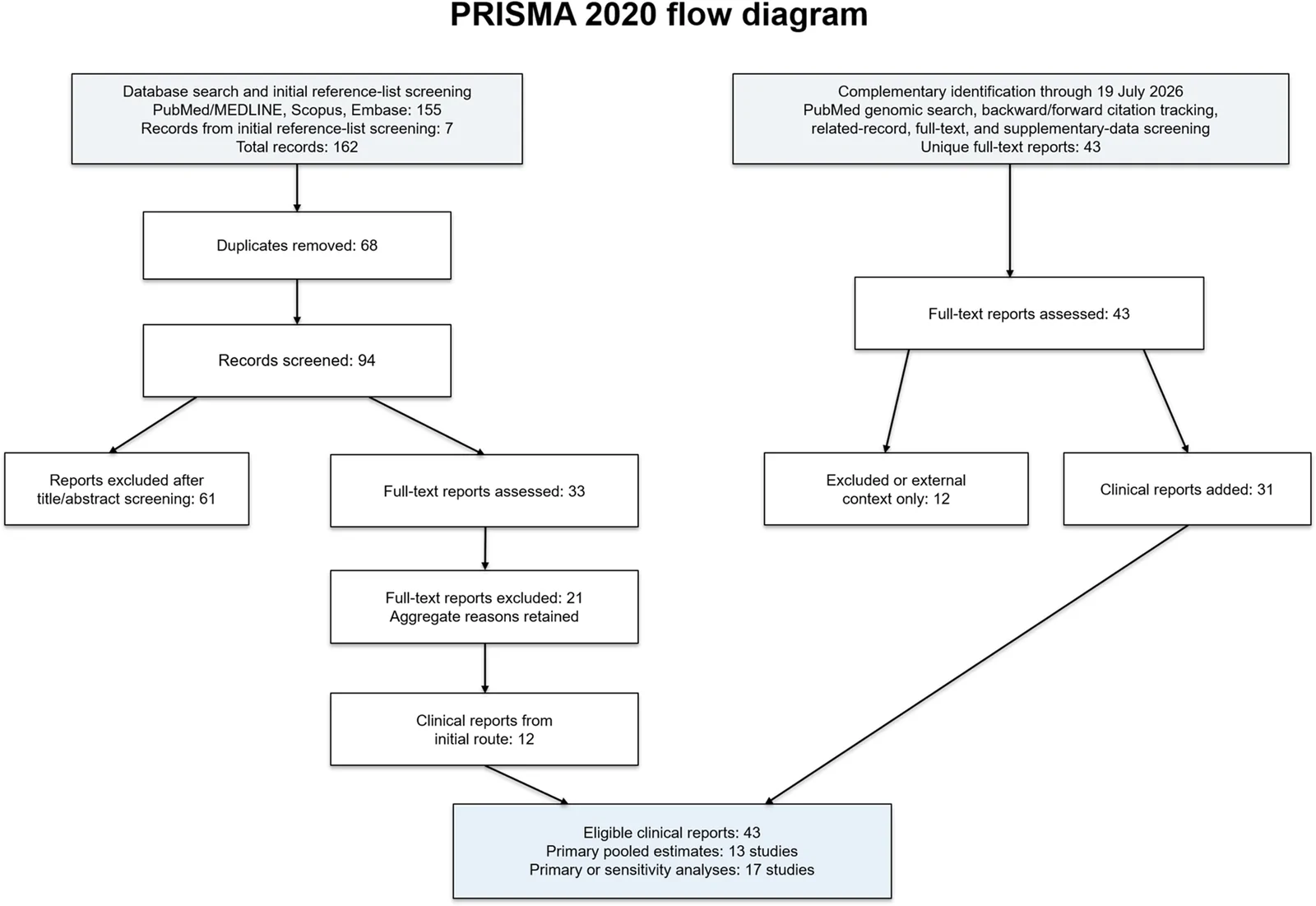
PRISMA 2020 flow diagram of study identification and selection through 19 July 2026. Forty-three eligible clinical reports were included; 13 contributed to a primary pooled estimate and 17 to at least one primary or sensitivity analysis

Records were indexed in a structured Microsoft Excel workbook and deduplicated by DOI, PMID, title, first author, publication year, and cohort provenance. Two investigators independently screened titles/abstracts and full texts, extracted data, and assigned endpoint eligibility; disagreements were resolved with a third investigator. Reporting followed PRISMA 2020 and PRISMA-S [33, 34]. No protocol was prospectively registered. Eligibility and overlap decisions for included reports are summarized in Table 1; aggregate exclusion counts are shown in Fig. 1.

**Table 1:** Characteristics and endpoint allocation of the 43 eligible clinical reports, ordered by publication year

Study	Setting	Use in review	Main applicability/overlap note
TCGA 2014	Clinical genomic PTC cohort	Qualitative	Outside quantitative endpoints
Kunstman 2015	ATC whole-exome cohort	ATC primary	Small genomic cohort
Jung 2016	Follicular adenoma/carcinoma WES	Qualitative/overlap context	Small Korean cohort; potentially linked to Jung 2018
Karunamurthy 2016	FNA and histologic tumor series	Pool 1/PDTC sensitivity; ATC primary	Source-defined cytology; PDTC criteria not explicit; linked pilot
Landa 2016	PDTC/ATC genomic cohort	PDTC and ATC primary analyses	8/52 Turin; historical sensitivity; MSK reference cohort
Yoo 2016	miFTC/FA/PTC genomic cohort	Qualitative	Histologic-spectrum context; same network as Yoo 2019, distinct cohort
Marques 2017	Familial nonmedullary thyroid carcinomas	Qualitative negative evidence	0/54 whole-gene *EIF1AX*; familial selection
Ganly 2018	Primary Hürthle-cell carcinomas	Qualitative	6/56; histology selected; MSK overlap assessment
Guan 2018	Consecutive indeterminate FNBs; *TSHR*/NIS focus	Qualitative molecular context	1/25 *TSHR*/NIS-altered nodules had *EIF1AX*/*GNAS*; complete 388-nodule *EIF1AX* numerator unavailable
Jung 2018	Follicular-patterned tumors and classic PTC	Qualitative	1/201 follicular-patterned tumors (follicular adenoma); 0/120 classic PTC
Nicolson 2018	WHO-classified follicular thyroid carcinomas	Qualitative/overlap context	2/39; Yale/Karolinska institutional family
Pozdeyev 2018	Advanced thyroid cancer	ATC sensitivity	Overlaps Landa
Alzahrani 2019	Unselected mixed thyroid carcinomas	Qualitative	1/274 assay-evaluable; positive histotype unavailable
Duan 2019, Diagn Pathol	Follicular tumor sequencing	Qualitative	Histologic-spectrum context
Duan 2019, Histopathology	Multicenter WHO-defined PDTC/ATC sequencing	PDTC and ATC primary analyses	2/41 PDTC; 2/25 ATC; independent Chinese cohort
Schatz-Siemers 2019	Hürthle-predominant Bethesda III/IV nodules	Qualitative	5/188; phenotype selected; one verified case benign
Simões-Pereira 2019	Mixed/progression PDTC/ATC	Sensitivity/qualitative	Selected for coexisting components
Yoo 2019	Advanced thyroid cancer sequencing	PDTC and ATC primary analyses	1/15 Turin-PDTC; 9/27 ATC; patient-level data
Kachko 2020	Mixed histologically verified thyroid lesions	Qualitative negative evidence	0/153; hotspot-restricted *EIF1AX* ascertainment
Desai 2021	415 Bethesda III/IV ThyroSeq v3 nodules; 127 histology	Broad Pool 2 primary	1/5 broad outcome; carcinoma versus NIFTP not separable; UPenn family
Gargano 2021	Indeterminate nodules	Pools 1 and 2; subgroups	Primary
Gilani 2021	Hürthle-cell neoplasm cytology	Qualitative/overlap context	5/85 *EIF1AX*-containing profiles; limited verification; probable Yale overlap
Bandargal 2022	*EIF1AX*-positive surgical cohort	Pool 2; subgroups	Bethesda V NIFTP excluded
Elsherbini 2022	Thyroidectomy-selected cohort	Pool 2 sensitivity	Linked McGill cohort
Gajzer 2022	541 AUS ThyroSeq v3 nodules; 155 surgical follow-up	Qualitative	Miami family; complete all-call *EIF1AX* numerator unavailable
Karslioglu French 2022	Operated *EIF1AX*-positive nodules	Pool 2; co-alterations	Strict BIII/IV after three exclusions
Xu 2022	Primary high-grade non-anaplastic carcinoma	Qualitative/overlap assessment	166 profiled; 76 previously reported; independent *EIF1AX* subset not separable
Alohali 2023	*TERT*-promoter-mutated surgical tumors	Qualitative molecularly enriched context	1/24 *PIK3CA*/*EIF1AX*; possible McGill overlap
Andrianus 2023	*TSHR*-mutated Bethesda III/IV nodules	Qualitative molecular context	3/69 with *EIF1AX*; *TSHR*-selected; no tissue outcome for *EIF1AX* subset
Tjendra 2023	92 Bethesda IV ThyroSeq v3 nodules	Qualitative	Miami family; source-internal *EIF1AX* count discrepancy
Abi-Raad 2024, YNHH	Bethesda III/IV molecular testing	Pools 1 and 2; subgroups	MSK referral subset excluded
Mejia-Mejia 2024	1226 AUS ThyroSeq-tested nodules	Qualitative	23 currently-negative *EIF1AX* calls; Miami quality-metric/overlap context
Oh 2024	Prospective panel cohort	Pool 1	3/115 in Bethesda III/IV
Payne 2024	203 resected Bethesda III/IV ThyroSeq v3 nodules	Qualitative	*EIF1AX* reported only within malignant/NIFTP outcomes; benign denominator absent; McGill overlap
Perry 2024	Negative/low-risk ThyroSeq Bethesda III/IV nodules	Qualitative low-risk outcome context	8/167 *EIF1AX*; 4 resected, 1 minimally invasive oncocytic FTC; result-selected
Sirotnikov 2024	276 Bethesda III-V ThyroSeq v3 nodules; 74 surgical follow-up	Qualitative	Three *EIF1AX*-containing profiles; patient-level outcome/category mapping unavailable; possible Emory overlap
Abi-Raad 2025, *TERT*-selected	*TERT*-positive FNA with histopathologic follow-up	Qualitative molecularly enriched context	4/32; one benign and three malignant; possible Yale overlap
Cocelli 2025	Oncocytic resections	Qualitative	Histology selected
Connelly 2025	Multiplatform BIII/IV cohort	Pool 2 broad only	1/2; strict malignancy not extractable
Odate 2025	Paired DTC-to-ATC progression	Qualitative	Progression selected
Pan 2025	PDTC/ATC proteogenomics	PDTC and ATC primary analyses	Primary; independent Chinese multicenter cohort
Scholfield 2025	WHO 2022 high-grade cohort	Qualitative; replacement sensitivity	PDTC/HGDTC mixed; MSK institutional overlap
Wong and Baloch 2026	*RAS*-positive Bethesda III-V ThyroSeq v3 nodules	Qualitative molecularly enriched context	21 *RAS*+*EIF1AX* cases; 11/21 malignant; *RAS*-selected; UPenn overlap assessed

*ATC* anaplastic thyroid carcinoma; *AUS* atypia of undetermined significance; *BIII/IV* Bethesda III/IV; *DTC* differentiated thyroid carcinoma; *FA* follicular adenoma; *FNA/FNB* fine-needle aspiration/biopsy; *FTC* follicular thyroid carcinoma; *HGDTC* high-grade differentiated thyroid carcinoma; *HGFCTC* high-grade follicular cell-derived non-anaplastic thyroid carcinoma; *miFTC* minimally invasive follicular thyroid carcinoma; *MSK/MSKCC* Memorial Sloan Kettering Cancer Center; *NGS* next-generation sequencing; *NIFTP* noninvasive follicular thyroid neoplasm with papillary-like nuclear features; *NIS* sodium-iodide symporter; *PDTC* poorly differentiated thyroid carcinoma; *PTC* papillary thyroid carcinoma; *TCGA* The Cancer Genome Atlas; *TSHR *thyroid-stimulating hormone receptor; *UPenn* University of Pennsylvania; *WES* whole-exome sequencing; *WHO* World Health Organization; *YNHH* Yale New Haven Hospital

The PubMed/MEDLINE strategy was: ("EIF1AX"[Title/Abstract] OR "eukaryotic translation initiation factor 1A X-linked"[Title/Abstract] OR "eIF1A"[Title/Abstract]) AND ("thyroid"[Title/Abstract] OR "thyroid nodule"[Title/Abstract] OR "thyroid cancer"[Title/Abstract] OR "thyroid carcinoma"[Title/Abstract] OR "papillary thyroid carcinoma"[Title/Abstract] OR "follicular thyroid carcinoma"[Title/Abstract] OR "poorly differentiated thyroid carcinoma"[Title/Abstract] OR "anaplastic thyroid carcinoma"[Title/Abstract] OR "Bethesda"[Title/Abstract] OR "fine needle aspiration"[Title/Abstract] OR "FNA"[Title/Abstract]).

The Scopus strategy was: TITLE-ABS-KEY (EIF1AX OR "eukaryotic translation initiation factor 1A X-linked" OR eIF1A) AND TITLE-ABS-KEY (thyroid OR "thyroid nodule" OR "thyroid cancer" OR "thyroid carcinoma" OR "papillary thyroid carcinoma" OR "follicular thyroid carcinoma" OR "poorly differentiated thyroid carcinoma" OR "anaplastic thyroid carcinoma" OR Bethesda OR "fine needle aspiration" OR FNA).

The Embase strategy was: ('eif1ax':ti,ab OR 'eukaryotic translation initiation factor 1a x-linked':ti,ab OR 'eif1a':ti,ab) AND ('thyroid':ti,ab OR 'thyroid nodule':ti,ab OR 'thyroid cancer':ti,ab OR 'thyroid carcinoma':ti,ab OR 'papillary thyroid carcinoma':ti,ab OR 'follicular thyroid carcinoma':ti,ab OR 'poorly differentiated thyroid carcinoma':ti,ab OR 'anaplastic thyroid carcinoma':ti,ab OR 'bethesda':ti,ab OR 'fine needle aspiration':ti,ab OR 'fna':ti,ab).

The supplementary PubMed/MEDLINE genomic strategy was: (thyroid[Title/Abstract] OR "thyroid nodule"[Title/Abstract] OR "follicular thyroid"[Title/Abstract] OR oncocytic[Title/Abstract] OR Hurthle[Title/Abstract] OR "poorly differentiated thyroid"[Title/Abstract] OR "anaplastic thyroid"[Title/Abstract] OR PDTC[Title/Abstract] OR ATC[Title/Abstract]) AND (genomic*[Title/Abstract] OR sequencing[Title/Abstract] OR "molecular profiling"[Title/Abstract] OR "mutation landscape"[Title/Abstract] OR proteogenomic*[Title/Abstract] OR "whole-exome"[Title/Abstract] OR "targeted panel"[Title/Abstract]). Equivalent concepts were used for citation and supplementary-data screening. Full texts, figures, oncoprints, and source-data tables were searched for EIF1AX.

### Eligibility by endpoint

Pool 1 evaluated the prevalence of any reported *EIF1AX* alteration among molecularly tested nodules with a strictly extractable Bethesda III/IV denominator and a documented, uniform opportunity to detect and report *EIF1AX*. Classifier-positive, low-level, or currently-negative calls were eligible only when the complete study-level *EIF1AX* numerator was reported for the entire eligible denominator. Karunamurthy et al. [7] was excluded from the primary analysis because its source-defined indeterminate denominator could not be separated from suspicious-for-malignancy cytology and was retained as a sensitivity analysis. Elsherbini et al. [11] was excluded because its denominator was nested within a thyroidectomy-selected population. Connelly et al. [35] combined molecular platforms without uniform *EIF1AX* ascertainment and was not pooled for prevalence. Three overlapping University of Miami reports were retained qualitatively: Gajzer [36] did not enumerate all currently-negative *EIF1AX* findings, Tjendra [37] contained irreconcilable study-level counts, and Mejia-Mejia [38] reported 23 currently-negative calls in an overlapping quality-metric cohort without an independent all-call cross-tabulation.

Pool 2 evaluated tissue-verified outcomes among *EIF1AX*-positive nodules. The primary malignant endpoint was restricted to FNA Bethesda III/IV. A secondary endpoint included malignant histology plus NIFTP or borderline follicular-patterned tumors. Karslioglu French contributed 9/28 malignant and 11/28 broad outcomes after exclusion of one Bethesda V ATC and two open-biopsy ATCs; Bandargal contributed 20/41 and 25/41 after exclusion of one Bethesda V NIFTP. Desai contributed 1/5 only to the broad endpoint because its molecular outcome table combined carcinoma and NIFTP [39]. Connelly likewise contributed 1/2 only to the broad endpoint [35]. Sirotnikov identified three *EIF1AX*-containing profiles but did not link each profile to individual histology or strict Bethesda III/IV status [40], and Payne reported *EIF1AX* only within malignant/NIFTP outcome groups without the benign *EIF1AX* denominator [41]; both were retained qualitatively. Bethesda III–V FNA and all-preoperative pathways were retained as sensitivities.

Pool 3 evaluated patient-level *EIF1AX* prevalence separately in WHO- or Turin-defined PDTC and in histology-defined ATC cohorts not molecularly preselected for *EIF1AX* or a specific co-driver. The primary PDTC analysis was restricted to studies that explicitly used WHO- or Turin-compatible criteria. Duan et al. [32] used the fourth-edition WHO classification; Landa [16] was restricted to 8/52 Turin-PDTC tumors; Yoo [42] explicitly used the Turin proposal; and Pan [19] used contemporary WHO-based classification. Karunamurthy [7] reported four source-defined PDTCs without explicit criteria and was retained only in sensitivity analysis. Historical institution-specific high-grade definitions, mixed high-grade follicular cell-derived non-anaplastic thyroid carcinoma (HGFCTC) cohorts, and progression-selected series were analyzed separately.

Beyond the three quantitative endpoints, qualitative synthesis included full-length English-language original clinical reports that directly described *EIF1AX* prevalence, genotype-phenotype associations, or negative *EIF1AX* findings in human thyroid nodules or follicular cell-derived thyroid tumors with an extractable study-level denominator. Molecularly or phenotypically enriched cohorts were eligible for qualitative synthesis but were not pooled with unselected target populations. Reviews, editorials, conference abstracts without sufficient cohort data, assay-development studies without clinically interpretable *EIF1AX* outcomes, single-case reports, preclinical studies without a clinical denominator, secondary reanalyses without an independent cohort, inseparable composite datasets, and pan-cancer datasets without separable thyroid-specific data were excluded from the eligible clinical-report count.

### Data extraction, subgroup coding, and cohort overlap

For each study, we extracted setting, recruitment period, cytologic and histologic eligibility, molecular platform, exact *EIF1AX* numerator and denominator, tissue outcome, variant class, co-alterations, source location, and overlap indicators. Source locations supporting data extraction and eligibility decisions were recorded for verification. Study-level values were checked against case-level tables and oncoprints where available. Yoo patient-level data were extracted from Supplementary Data 2: one of 15 Turin-PDTCs and nine of 27 ATCs harbored *EIF1AX* [42].

Splice-site analyses contrasted alterations involving the recurrent A113 splice region with non-splice variants. Co-alteration analyses contrasted isolated *EIF1AX* with *EIF1AX* accompanied by at least one additional pathogenic/reportable alteration. Karslioglu French was excluded from the strict splice analysis because the variant class of the three excluded ATCs could not be mapped with certainty. The Bandargal Bethesda V NIFTP was non-splice and isolated and was removed from the corresponding denominators. Because only three or four independent study clusters were available, subgroup pooled proportions were treated descriptively and formal interaction tests were not used for primary interpretation. Repeated-denominator PDTC/ATC isolated-versus-co-mutated models were not fitted.

Karunamurthy [7] and Karslioglu French [10] were not included together in a primary tissue-outcome estimate because three later cases had appeared in the pilot. Elsherbini [11], Bandargal [12], Bourque [43], Alohali [44], and Payne [41] were treated as a linked McGill family. Gajzer [36], Tjendra [37], and Mejia-Mejia [38] were treated as a linked University of Miami family. Desai [39], Andrianus [45], and Wong and Baloch [21] were treated as a linked UPenn family; the three *TSHR*-plus-*EIF1AX* cases in Andrianus lacked surgery and therefore did not duplicate the five operated Desai cases used in the broad Pool 2 analysis; Wong and Baloch was *RAS*-selected and retained only as qualitative evidence because patient-level independence from the other UPenn cohorts could not be established. Sirotnikov [40] and the Emory component of Abi-Raad [46] were treated as potentially linked. Landa [16], Xu [31], Scholfield [47], Pozdeyev [18], and the clinical-genomic component of Krishnamoorthy [22] belong to a linked MSK advanced/high-grade family and were not pooled concurrently when patient-level independence could not be established. The principal applicability and overlap decisions are summarized in Table 1.

Within the linked UPenn family, Wong and Baloch [21] selected nodules for *RAS* positivity; its 21 *RAS*-plus-*EIF1AX* cases were retained as molecularly enriched qualitative evidence and were not mixed with unselected Pool 2 cohorts. Schatz-Siemers [48] and Gilani [49] were restricted to Hürthle-cell-predominant cytology and were retained as phenotype-specific qualitative evidence. Ganly [23], Nicolson [24], Jung [25], Marques [26], Yoo [27], and Jung [28] extended the histologic spectrum but did not match the target populations of the five primary proportion estimates. Kachko [50] contributed negative evidence from a mixed histologic cohort with hotspot-restricted ascertainment. Alohali [44] and Abi-Raad [46] were selected for *TERT* promoter mutation and were retained as molecularly enriched context. Cocelli [51] and Odate [52] were likewise retained as phenotype- or progression-specific qualitative evidence.

Additional molecular-result-selected or source-ambiguous indeterminate-nodule cohorts were retained only in qualitative synthesis. Guan [53] evaluated 388 consecutive indeterminate FNBs but reported *EIF1AX* only within the *TSHR*/NIS-altered subset. Andrianus [45] identified three *EIF1AX* co-mutations among 69 *TSHR*-mutated nodules without tissue verification of those cases. Perry [54] followed eight low-risk *EIF1AX* findings in a negative/low-risk-selected cohort. Gajzer [36], Tjendra [37], and Mejia-Mejia [38] documented classifier-positive or currently-negative *EIF1AX* findings in overlapping Miami cohorts but did not provide a single reconcilable, independent all-event denominator. Sirotnikov [40] reported three *EIF1AX*-containing profiles without patient-level outcome mapping, and Payne [41] reported 22 *EIF1AX* findings only among malignant/NIFTP outcomes. These selection or reporting mechanisms precluded pooling with the unselected primary populations.

### Risk-of-bias and applicability appraisal

Risk of bias was assessed with the Joanna Briggs Institute checklist for prevalence studies, adapted to molecular and surgical pathology [55, 56]. Domains addressed sampling frame, selection, endpoint precision, setting, denominator coverage, molecular ascertainment, histopathologic verification, analysis, and completeness of verification/follow-up. Judgments were endpoint specific, reasons for No/Unclear ratings were recorded, and no numerical summary score was used. Formal endpoint-specific JBI judgments for studies contributing to primary or sensitivity analyses are reported in Table 3. Qualitative-only reports were assessed narratively for applicability, as summarized in Table 1.

### Statistical analysis

All statistical analyses were performed in R version 4.6.1 (R Foundation for Statistical Computing, Vienna, Austria).

**Fig. 2:**
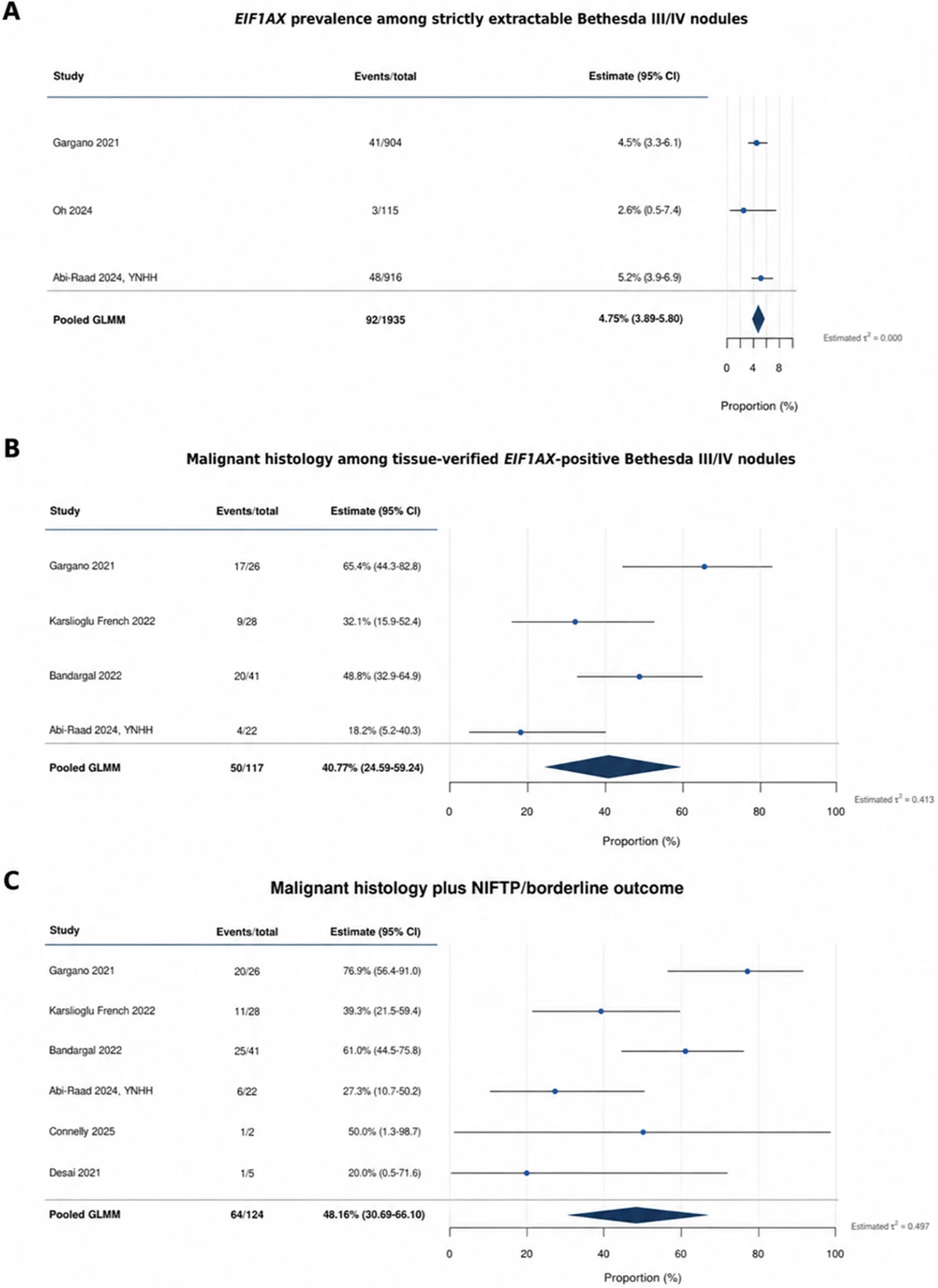
Integrated study tables and forest plots for indeterminate-nodule endpoints: (**A**) EIF1AX prevalence among strictly extractable Bethesda III/IV nodules; (**B**) strict malignant histology among tissue-verified EIF1AX-positive Bethesda III/IV nodules; and (**C**) malignant histology plus NIFTP/borderline outcome. Study intervals are exact Clopper–Pearson intervals; pooled estimates are binomial-normal GLMMs

**Fig. 3:**
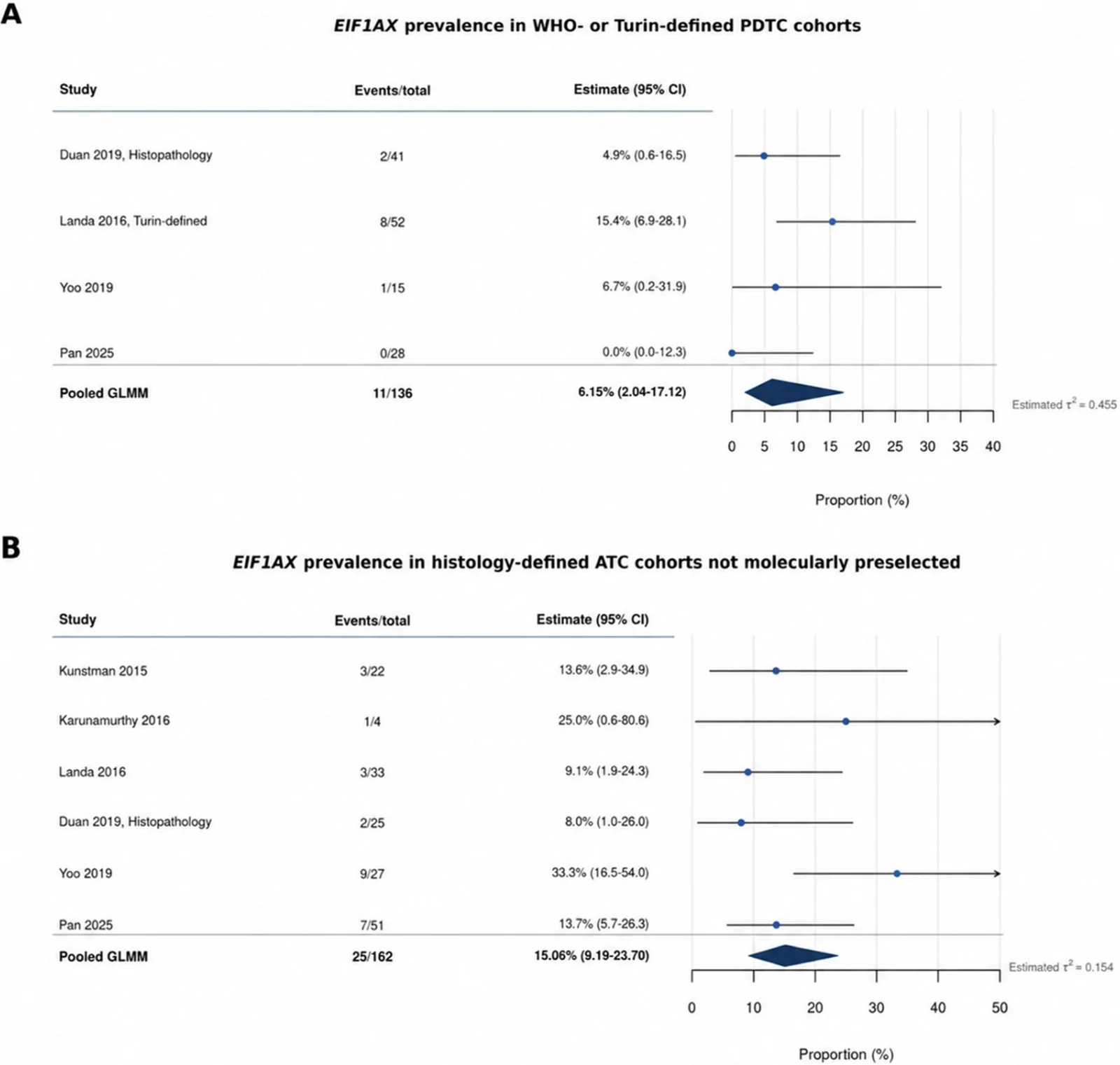
Integrated study tables and forest plots for advanced carcinoma: (**A**) WHO- or Turin-defined PDTC; and (**B**) histology-defined ATC cohorts not molecularly preselected. Source-defined, progression-selected, historical, mixed high-grade, and overlapping cohorts were excluded from the primary estimates and evaluated separately. In panel B, confidence intervals extending beyond the plotting range are truncated and indicated by arrows; complete limits are printed in the study table

Pooled proportions were estimated directly from events and denominators using binomial-normal generalized linear mixed models with a logit link, random study intercept, maximum-likelihood estimation, and 15-point adaptive Gauss-Hermite quadrature (metafor::rma.glmm; measure="PLO"). No transformed inverse-variance method or continuity correction was used. Individual-study 95% confidence intervals were exact Clopper-Pearson intervals. Forest plots were generated from the R output and are presented in Figures 2 and 3; Figure 1 summarizes the PRISMA study-selection process.

Between-study variance was reported as τ² on the logit scale. A boundary estimate of zero was not interpreted as proof of homogeneity. Leave-one-out and planned replacement analyses assessed stability. Subgroup pooled proportions were descriptive; formal interaction P values were omitted because three or four clusters cannot support reliable asymptotic inference.

Funnel plots and Egger-type tests were not performed. In a one-group meta-analysis of proportions, the relationship between sampling variance and observed proportion is structural, and conventional funnel asymmetry methods do not provide a valid publication-bias assessment; the small number of studies further limits interpretation.

## Results

### Study selection and study characteristics

The database search and initial reference-list screening identified 162 records. After removal of 68 duplicates, 94 records were screened; 61 were excluded at title/abstract review, leaving 33 full-text reports for assessment. Of these, 21 were excluded and 12 entered the initial clinical evidence base. Complementary genomic, citation, related-record, full-text, and supplementary-data searches subsequently identified 43 unique full-text reports for detailed assessment; 31 additional clinical reports met the eligibility criteria, whereas 12 were excluded or retained only as external, technical, case-report, secondary-analysis, mechanistic, or overlap context. The final evidence base therefore comprised 43 eligible clinical reports. Thirteen contributed to at least one primary pooled estimate, 17 to at least one primary or sensitivity analysis, and 26 contributed only to qualitative synthesis (Fig. 1). Study characteristics, endpoint allocation, and the principal applicability decisions are summarized in Table 1.

### Prevalence among molecularly tested indeterminate nodules

Three studies had a strictly extractable Bethesda III/IV denominator, a complete study-level *EIF1AX* numerator, and compatible ascertainment [9, 13, 14]. Across 1935 tested nodules, 92 harbored *EIF1AX*. The pooled prevalence was 4.75% (95% CI, 3.89%–5.80%; τ²=0.000). Adding the source-defined Karunamurthy cohort in sensitivity analysis produced 119/2582 and 4.61% (95% CI, 3.86%–5.49%; τ²=0.000). Gajzer, Tjendra, and Mejia-Mejia were not pooled because the overlapping Miami sources did not provide a single independent, reconcilable numerator for all reported *EIF1AX* call categories [36–38]. Connelly 7/1252 was not pooled because the denominator combined assays without uniform *EIF1AX* coverage/reporting (Fig. 2 A; Tables 1 and 2).

**Table 2:** Primary, subgroup, and sensitivity meta-analytic results

Category	Analysis	k	Events/total	Estimate	95% CI	*τ*²
Primary	Strict Bethesda III/IV prevalence	3	92/1935	4.75%	3.89–5.80%	0.000
Primary	Strict malignant histology	4	50/117	40.77%	24.59–59.24%	0.413
Primary	Malignant + NIFTP/borderline	6	64/124	48.16%	30.69–66.10%	0.497
Primary	WHO- or Turin-defined PDTC	4	11/136	6.15%	2.04–17.12%	0.455
Primary	ATC cohorts not molecularly preselected	6	25/162	15.06%	9.19–23.70%	0.154
Subgroup	Malignant histology: splice-site	3	28/52	52.35%	16.80–85.67%	1.885
Subgroup	Malignant histology: non-splice	3	13/37	35.14%	21.62–51.55%	0.000
Subgroup	Broad endpoint: splice-site	3	31/52	60.95%	28.01–86.23%	1.191
Subgroup	Broad endpoint: non-splice	3	20/37	54.05%	38.13–69.19%	0.000
Subgroup	Malignant histology: isolated *EIF1AX*	4	19/65	27.18%	14.84–44.42%	0.146
Subgroup	Malignant histology: co-mutated *EIF1AX*	4	31/52	61.50%	32.87–83.90%	1.042
Subgroup	Broad endpoint: isolated *EIF1AX*	4	25/65	32.20%	14.10–57.88%	0.711
Subgroup	Broad endpoint: co-mutated *EIF1AX*	4	37/52	75.10%	46.96–91.13%	1.020
Sensitivity	Pool 1 + source-defined Karunamurthy	4	119/2582	4.61%	3.86–5.49%	0.000
Sensitivity	Malignant, Bethesda III–V FNA	4	51/119	41.15%	25.39–58.96%	0.376
Sensitivity	Broad, Bethesda III–V FNA	6	66/126	48.90%	31.44–66.62%	0.487
Sensitivity	Malignant, all preoperative pathways	4	53/121	42.34%	26.89–59.46%	0.340
Sensitivity	Broad, all preoperative pathways	6	68/128	49.84%	32.64–67.08%	0.456
Sensitivity	Malignant, Karunamurthy replaces Karslioglu	4	42/94	39.91%	20.99–62.41%	0.550
Sensitivity	Broad, Karunamurthy replaces Karslioglu	6	54/101	45.81%	25.45–67.67%	0.696
Sensitivity	Malignant, Elsherbini replaces Bandargal	4	42/107	37.98%	22.69–56.10%	0.384
Sensitivity	Broad, Elsherbini replaces Bandargal	6	53/114	44.54%	28.47–61.84%	0.422
Sensitivity	Malignant, exclude linked families	2	21/48	40.08%	12.59–75.64%	1.000
Sensitivity	Broad, exclude linked families	4	28/55	44.92%	19.34–73.50%	0.909
Sensitivity	PDTC + source-defined Karunamurthy	5	11/140	5.75%	1.83–16.67%	0.477
Sensitivity	PDTC + progression-selected Simões-Pereira	5	13/145	7.70%	3.22–17.30%	0.377
Sensitivity	Historical high-grade including Landa MSKCC-only	4	12/168	6.50%	2.61–15.27%	0.090
Sensitivity	High-grade, Scholfield replaces Landa	4	15/190	6.55%	2.46–16.29%	0.165
Sensitivity	ATC + progression-selected Simões-Pereira	7	25/169	14.06%	8.37–22.65%	0.177
Sensitivity	ATC, Pozdeyev replaces Landa	6	24/160	14.39%	8.34–23.72%	0.220

*GLMM* generalized linear mixed model. Between-study variance is τ² on the logit scale. Subgroup estimates are descriptive and exploratory; formal interaction P values were not used for primary interpretation. Variant-class analyses included Gargano [9], Bandargal [12], and the Abi-Raad YNHH cohort [13]; co-alteration analyses additionally included Karslioglu French [10]. Study-level subgroup data are available with the analytic files. Broad Bethesda III–V and all-preoperative sensitivity analyses retain the outcome-compatible Desai and Connelly contributions used in the primary broad endpoint

### Tissue-verified outcomes among *EIF1AX*-positive nodules

Four non-overlapping cohorts contributed to the strict malignant tissue-outcome analysis [9, 10, 12, 13]. After removing the Karslioglu Bethesda V/open-biopsy ATCs and the Bandargal Bethesda V NIFTP from the eligible denominators, malignant histology occurred in 50/117 nodules. The pooled proportion was 40.77% (95% CI, 24.59%–59.24%; τ²=0.413). These data describe surgically selected, tissue-verified nodules and are not a universal probability for all *EIF1AX*-positive FNA results (Fig. 2B; Table 2).

For malignant histology plus NIFTP/borderline outcome, Desai contributed 1/5 and Connelly contributed 1/2 in addition to the four strict cohorts, yielding 64/124. The pooled proportion was 48.16% (95% CI, 30.69%–66.10%; τ²=0.497). Neither study could enter the strict malignant endpoint because its molecular outcome category combined carcinoma with NIFTP or an equivalent borderline grouping (Fig. 2C; Table 2).

### Variant class and co-alteration profile

Strict outcome-specific splice data were available from three cohorts. The pooled malignant proportions were 52.35% for splice variants (95% CI, 16.80%–85.67%; τ²=1.885) and 35.14% for non-splice variants (95% CI, 21.62%–51.55%; τ²=0.000). For the broad endpoint, the corresponding estimates were 60.95% (95% CI, 28.01%–86.23%; τ²=1.191) and 54.05% (95% CI, 38.13%–69.19%; τ²=0.000). The interval widths preclude a categorical splice-effect claim (Table 2).

Co-alteration status showed a larger descriptive separation. The pooled malignant proportion was 27.18% for isolated *EIF1AX* (95% CI, 14.84%–44.42%; τ²=0.146) and 61.50% for co-mutated profiles (95% CI, 32.87%–83.90%; τ²=1.042). Broad-outcome estimates were 32.20% (95% CI, 14.10%–57.88%; τ²=0.711) and 75.10% (95% CI, 46.96%–91.13%; τ²=1.020). These are hypothesis-generating pooled descriptions, not validated prognostic cutoffs (Table 2).

Wong and Baloch reported 11/21 malignant outcomes in a *RAS*-selected *RAS*-plus-*EIF1AX* cohort [21]. This directionally supports integrated co-driver interpretation but was not pooled with unselected studies. Pool 1 lacked compatible tested-population denominators by variant class or co-alteration status.

### Risk of bias and applicability

Across Pool 1, molecular ascertainment and denominator coverage were generally adequate in the three primary cohorts, but sample-size or precision rationales were usually absent and most cohorts were retrospective. The overlapping Miami reports had additional applicability concerns because classifier-positive and currently-negative *EIF1AX* calls were incompletely or inconsistently cross-tabulated. Pool 2 had the greatest risk of selection and verification bias because surgery and tissue availability conditioned inclusion; Desai and Connelly also grouped carcinoma with NIFTP in the relevant molecular outcome table. Sirotnikov lacked patient-level mapping of three *EIF1AX*-containing profiles, and Payne lacked a benign *EIF1AX* denominator. Advanced-carcinoma cohorts were small, referral or research-tissue selected, historically classified, or progression enriched. These considerations informed the separation of primary, sensitivity, and qualitative analyses; endpoint-adapted JBI judgments for the quantitative evidence are shown in Table 3.

**Table 3:** Endpoint-adapted Joanna Briggs Institute appraisal of studies contributing to primary or sensitivity analyses

Study/endpoint	D1–D3	D4–D6	D7–D9	Key concern
Karunamurthy—Pool 1 sensitivity	U/U/U	Y/N/Y	NA/Y/Y	Mixed/source-defined cytology
Karunamurthy—Pool 2 pilot	N/N/U	Y/Y/Y	Y/Y/N	Only 5/27 verified
Karunamurthy—advanced sensitivity	N/U/U	Y/Y/Y	Y/Y/Y	Very small subsets; PDTC criteria not explicit
Gargano—Pool 1	Y/Y/U	Y/Y/Y	NA/Y/Y	Retrospective
Oh—Pool 1	Y/Y/U	Y/Y/Y	NA/Y/Y	Three events
Abi-Raad 2024 YNHH—Pool 1	Y/Y/U	Y/Y/Y	NA/Y/Y	Institution-dependent testing
Gargano—Pool 2	U/U/U	Y/Y/Y	Y/Y/U	Verification selection
Karslioglu—Pool 2	N/N/U	Y/Y/Y	Y/Y/Y	Operated positive cohort
Bandargal—Pool 2	N/N/U	Y/Y/Y	Y/Y/Y	Selected surgical cohort
Abi-Raad 2024 YNHH—Pool 2	N/N/U	Y/Y/Y	Y/Y/U	Surgical selection
Connelly—broad Pool 2	N/N/U	Y/N/U	Y/Y/N	Only 2/7 verified; outcome grouped
Elsherbini—sensitivity	N/N/U	Y/Y/Y	Y/Y/Y	Thyroidectomy-selected
Duan Histopathology—PDTC/ATC	U/U/U	Y/Y/Y	Y/Y/Y	Multicenter retrospective; 18-gene panel
Landa—PDTC/ATC	U/U/U	Y/Y/Y	Y/Y/Y	Referral/high-grade enrichment; overlap family
Kunstman—ATC	U/U/U	Y/Y/Y	Y/Y/Y	Small genomic cohort
Pan—PDTC/ATC	U/U/U	Y/Y/Y	Y/Y/Y	Research-tissue cohort
Scholfield—HGFCTC sensitivity	N/U/U	Y/N/Y	Y/Y/N	46% sequenced; mixed histology; overlap
Yoo—PDTC/ATC	U/U/U	Y/Y/Y	Y/Y/Y	Advanced-disease sequencing; mixed specimen sites
Simões-Pereira—progression sensitivity	N/N/U	Y/Y/Y	Y/Y/Y	Progression-selected; very small series
Pozdeyev—ATC replacement	U/U/U	Y/Y/U	Y/Y/Y	Registry/clinical sequencing; overlaps Landa
Desai—broad Pool 2	N/N/U	Y/Y/Y	Y/Y/U	Surgery-selected; carcinoma/NIFTP grouped

*Domains D1* representative sampling frame; *D2* appropriate selection; *D3* sample size or endpoint precision justified; *D4* setting/participants described; *D5* eligible denominator covered; *D6* valid and uniform molecular testing; *D7* valid histopathologic verification when required; *D8* appropriate analysis; *D9* adequate response, follow-up, or verification. *Y* yes; *N* no; U unclear; *NA* not applicable. No numerical summary score was used. Formal JBI judgments are shown for studies contributing to primary or sensitivity analyses; qualitative-only reports were assessed narratively for applicability, as summarized in Table 1

### *EIF1AX* prevalence in advanced thyroid carcinoma

The primary PDTC analysis comprised four cohorts with explicit WHO- or Turin-compatible classification [16, 19, 32, 42]. Duan contributed 2/41, Landa contributed 8/52 Turin-PDTC tumors, Yoo contributed 1/15, and Pan contributed 0/28. Across 136 tumors, 11 were *EIF1AX*-positive; the pooled prevalence was 6.15% (95% CI, 2.04%–17.12%; τ²=0.455). The estimate remained imprecise. Adding the four source-defined Karunamurthy PDTCs yielded 5.75% (95% CI, 1.83%–16.67%), whereas adding the progression-selected Simões-Pereira cohort yielded 7.70% (95% CI, 3.22%–17.30%). Historical high-grade and Scholfield replacement sensitivities are reported separately (Fig. 3A; Table 2).

Six histology-defined ATC cohorts not molecularly preselected for *EIF1AX* or a specific co-driver reported 25 *EIF1AX*-positive tumors among 162 tumors [7, 16, 17, 19, 32, 42]. The pooled prevalence was 15.06% (95% CI, 9.19%–23.70%; τ²=0.154). Adding the progression-selected Simões-Pereira cohort yielded 14.06% (95% CI, 8.37%–22.65%). Replacing Landa with the overlapping Pozdeyev subset yielded 14.39% (95% CI, 8.34%–23.72%). Odate was not pooled because cases were selected for a coexisting differentiated component (Fig. 3B; Table 2).

### Sensitivity analyses

Primary, subgroup, and sensitivity results are summarized in Table 2. Sensitivity analyses broadened cytology to Bethesda III–V FNA, included all preoperative pathways, replaced linked cohort families, substituted source-defined, historical, or mixed high-grade definitions, and added progression-selected cohorts separately. Inclusion of Desai lowered the broad primary estimate from 51.68% to 48.16%; the corresponding broad sensitivity estimates remained directionally similar but continued to show substantial imprecision. Leave-one-out estimates ranged from 4.32% to 4.95% for Pool 1, 33.92% to 48.52% for malignant histology, 41.49% to 54.54% for the broad outcome, 3.57% to 9.79% for PDTC, and 11.85% to 16.76% for ATC. None supports a universal tissue-outcome probability. The PDTC estimate remained imprecise, while the addition of Yoo introduced measurable between-study variance in the ATC pool.

## Discussion

### Principal findings

The strict primary estimate indicates that *EIF1AX* is detected in approximately 4.75% of molecularly tested Bethesda III/IV nodules. The 4.61% source-defined sensitivity analysis, which included Karunamurthy [7], illustrates the influence of denominator definition and ascertainment. Cohorts with nonuniform *EIF1AX* ascertainment, incomplete reporting, or unresolved overlap were retained qualitatively rather than incorporated into the primary estimate [35–38].

Among *EIF1AX*-positive Bethesda III/IV nodules selected for tissue verification, the pooled malignant proportion was 40.77%. The broad malignant-plus-NIFTP/borderline estimate was 48.16% after adding the outcome-compatible Desai and Connelly data. Desai contributed one event among five operated *EIF1AX*-positive nodules, but its molecular table grouped carcinoma and NIFTP [39]. Verification, referral, and surgery-selection biases remain substantial, and neither pooled value is a universal preoperative probability.

The advanced-carcinoma results depended strongly on histologic definition, platform, and cohort independence. Incorporation of the WHO-defined Duan cohort and the Turin-defined Yoo cohort increased the primary PDTC evidence base to 136 tumors and the ATC evidence base to 162 tumors. Landa contributed 8/52 Turin-PDTC tumors; the ninth *EIF1AX*-positive case belonged to the MSKCC-only high-grade group. Yoo contributed one *EIF1AX*-positive Turin-PDTC and nine *EIF1AX*-positive ATCs from patient-level supplementary data [42]. Xu and Scholfield could not be treated as independent additions because both arose from the extended MSK high-grade cohort family. WHO- or Turin-defined PDTC prevalence remained imprecise and study dependent; prevalence in histology-defined ATC cohorts not molecularly preselected was approximately 15% and showed measurable estimated between-study variance.

### Molecular and histopathologic interpretation

*EIF1AX* is best regarded as a context-dependent event. Isolated non-splice alterations were more often associated with benign or low-risk follicular-patterned lesions, although malignant non-splice tumors were also reported. Co-mutated profiles were more frequently associated with invasive or high-grade disease [7–13, 21, 22]. The descriptive separation by co-alteration status was larger than that by splice class, but neither establishes causality.

A working biological model is that *EIF1AX* can provide an early permissive state while additional MAPK-pathway or progression-associated events contribute to transformation. Experimental cooperation between *EIF1AX* and *RAS* supports this hypothesis [22]. In Yoo, all nine *EIF1AX*-positive ATCs and the single *EIF1AX*-positive PDTC were identified from patient-level data, and most carried a *RAS*-family co-driver [42]. In paired FTC-to-ATC samples, Odate observed retention of *EIF1AX* with *HRAS* and *TERT* in two progression-selected tumors [52]. *TERT* and *TP53* remain independent progression-associated signals rather than consequences of *EIF1AX* itself [16–18, 29, 30].

Histologic classification remains indispensable. NIFTP, tumors of uncertain malignant potential, follicular adenoma, oncocytic neoplasia, differentiated carcinoma, HGDTC, WHO- or Turin-defined PDTC, and ATC cannot be collapsed into one endpoint [23, 31, 32, 47, 51, 57–60]. Xu reported higher *EIF1AX* frequency in HGTC-PDTC than HGTC-nonPDTC but included a substantial previously published molecular subset [31]. Scholfield reported *EIF1AX* in 13/117 sequenced high-grade follicular cell-derived tumors or neoplasms, but the series combined PDTC, HGDTC, and noninvasive lesions and overlapped the MSK institutional family [47].

Histology-selected and familial cohorts further document the breadth of the *EIF1AX* phenotype without altering the five primary pooled estimates. Ganly identified *EIF1AX* in 6/56 Hürthle-cell carcinomas [23], and Nicolson found recurrent *EIF1AX* mutations in 2/39 follicular carcinomas spanning WHO invasion subtypes [24]. Jung detected a single *EIF1AX*-mutated follicular adenoma among 201 follicular-patterned tumors and none among 120 classic papillary carcinomas [25], whereas Marques found no *EIF1AX* mutation after sequencing the whole gene in 54 familial nonmedullary thyroid carcinomas [26]. Earlier Korean genomic cohorts also detected *EIF1AX* within the follicular adenoma/minimally invasive follicular carcinoma spectrum [27, 28]; potential institutional linkage prevented their use as independent quantitative contributions. The clinical-genomic dataset in Krishnamoorthy combined an institutional series with Landa and Kunstman and was therefore retained only for mechanistic and overlap context [22].

### Clinical implications, cytopathology context, and generalizability

An *EIF1AX*-positive result should modify, not replace, the integrated assessment of cytology, sonography, patient factors, and the complete molecular profile. Cytologic molecular-profiling studies illustrate how specimen preparation, panel breadth, assay generation, and institutional workflow shape the information produced [1, 2, 35].

A molecular result labeled negative or low risk also requires longitudinal clinicopathologic interpretation. In Perry et al. [54], eight of 167 negative or low-risk ThyroSeq nodules carried *EIF1AX* findings; four were resected, and an isolated splice-site *EIF1AX* nodule was a minimally invasive oncocytic follicular carcinoma. This selected cohort does not provide a general malignancy probability, but it illustrates that assay-generation-specific risk labels should not replace surveillance and morphology.

Computational decision-support approaches have been explored for indeterminate thyroid nodules [5]. Studies of cystic specimens, rosette-forming papillary carcinoma, psammoma bodies, and ancillary markers similarly show that an isolated feature acquires diagnostic weight only within a representative, morphologically defined, and technically controlled specimen [61–66].

Phenotype-selected and institutional ThyroSeq series further illustrate this limitation. Schatz-Siemers identified *EIF1AX* in 5/188 Hürthle-predominant Bethesda III/IV nodules; the only *EIF1AX*-positive case with surgical follow-up was benign [48]. Gilani reported five *EIF1AX*-containing profiles among 85 tested Hürthle-cell neoplasms; three had tissue follow-up, but the cohort probably overlaps the later Abi-Raad YNHH series [49]. Cocelli reported three A113-splice alterations among 11 oncocytic carcinomas [51]. Gajzer reported a benign oncocytic adenoma with a high-level *EIF1AX* finding [36], whereas Payne found 22 *EIF1AX* alterations among malignant/NIFTP outcomes but did not report the benign *EIF1AX* denominator and substantially overlapped other McGill cohorts [41]. None of these observations can be converted into a general preoperative probability.

Thyroid cancers show substantial biological and clinical heterogeneity [67, 68]. Alzahrani identified one A113-splice *EIF1AX* alteration among 274 assay-evaluable tumors in a 301-case unselected thyroid-carcinoma cohort, but the histologic category of the positive tumor was not reported [69]. Panels differ in gene content, version, variant thresholds, copy-number and fusion detection, and whether specific variants are reported. Surgical referral thresholds and histopathologic practice also differ. The pooled tissue-verified estimates apply only to the source settings and should not be presented as universal predictive values. Current ATA guidelines emphasize risk-adapted multidisciplinary decision-making [70, 71].

Additional selected or assay-limited reports further reinforce the importance of defining the target population [38, 40, 44–46, 50, 53, 54]. Because these cohorts were molecularly or phenotypically selected, incompletely mapped, assay restricted, and/or potentially overlapping, they were retained as qualitative evidence and were not pooled with the unselected endpoint populations.

An isolated *EIF1AX* result should not by itself trigger aggressive treatment. Conversely, *EIF1AX* accompanied by *RAS*, *TERT*, *TP53*, or multiple additional alterations warrants greater concern and multidisciplinary review. In ATC, pathology-focused syntheses, registry studies, and clinical guidelines place ancillary biomarkers within the broader context of stage, actionable drivers, and multidisciplinary treatment [70, 72, 73]. Precision-oncology approaches focus on established actionable alterations such as *BRAF* V600E and *NTRK* fusions; *EIF1AX* remains an interpretive and hypothesis-generating marker rather than an established therapeutic target.

### Strengths and limitations

Strengths of this systematic review and meta-analysis include endpoint-specific eligibility criteria, complementary database and genomic-identification strategies, direct binomial GLMMs with exact study-level intervals and explicit τ² reporting, and conservative management of potentially overlapping cohorts. Where available, data were extracted at the patient, case, or table level, and strict and broader cytologic and histologic pathways were analyzed separately. Both positive and negative qualitative evidence was retained when informative, and the five endpoint-specific study-table forest plots were generated from the R analyses and presented in two composite figures.

Limitations remain substantial. Most cohorts were retrospective; Pool 2 was conditioned on tissue verification; advanced cohorts were small and sometimes research-tissue selected. Platforms, classifier call categories, reporting thresholds, and histologic terminology changed over time, and subgroup cross-tabulations were incomplete. Some overlapping or incompletely reported cohorts did not provide fully independent denominators or patient-level cross-tabulation of molecular findings, cytologic category, and histologic outcome. Database indexing, restriction to English-language full texts, complementary search methods, and absence of prospective protocol registration may have introduced selection bias. The WHO- or Turin-defined PDTC analysis remained imprecise and sensitive to study definition. Because subgroup analyses included few independent study clusters, their pooled proportions should be considered exploratory.

Future studies should report the complete assay-eligible denominator, Bethesda category, exact *EIF1AX* variant, splice status, all co-alterations, platform/version and reporting threshold, tissue-verification rate, current WHO histology, overlap identifiers, and follow-up. Prospective consecutive cohorts with outcome ascertainment independent of the molecular result are needed. For high-grade tumors, *EIF1AX* status must be cross-tabulated separately for Turin-PDTC, HGDTC, noninvasive high-grade lesions, and ATC.

## Conclusion

*EIF1AX* was detected in 4.75% of strictly extractable Bethesda III/IV nodules. Among selected *EIF1AX*-positive Bethesda III/IV nodules with tissue verification, 40.77% had malignant histology and 48.16% met the malignant-plus-NIFTP/borderline endpoint. These are not universal predictive values. Prevalence in WHO- or Turin-defined PDTC was 6.15% but remained imprecise and study dependent, whereas prevalence in histology-defined ATC cohorts not molecularly preselected was 15.06%. Variant class, histologic setting, assay, reporting threshold, and especially co-alteration architecture must be integrated into interpretation.

## Data Availability

Study-level data underlying the five primary pooled analyses are displayed in Figs. 2 and 3. Complete primary, subgroup, and sensitivity estimates are reported in Table 2; the study-level subgroup dataset, R code, and additional analytic files are available from the corresponding author on reasonable request.
